# Quality of Life of Parents of Premature Infants: A Systematic Review and Meta-Analysis

**DOI:** 10.1001/jamanetworkopen.2025.53712

**Published:** 2026-01-14

**Authors:** Sarah Angelique Shi En Yip, Queena Zhi Xuan Lim, Gwyneth Kong, Zubair Amin, Yvonne Peng Mei Ng

**Affiliations:** 1 Lee Kong Chian School of Medicine, Nanyang Technological University, Singapore; 2 Department of Medicine, National University Hospital, Singapore; 3 Department of Paediatrics, Yong Loo Lin School of Medicine, National University of Singapore, Singapore; 4 Department of Neonatology, Khoo Teck Puat-National University Children’s Medical Institute, National University Hospital, Singapore

## Abstract

**Importance:**

Quality of life (QOL) of parents following the birth of a premature infant is substantially affected, necessitating a comprehensive understanding of its determinants to guide effective interventions.

**Objectives:**

To systematically assess QOL among parents of premature infants and to identify modifiable factors associated with their well-being.

**Data Sources:**

PubMed, CINAHL, and Embase were searched from inception to October 5, 2025.

**Study Selection:**

Cross-sectional studies, longitudinal cohort studies, and randomized clinical trials reporting quantitative QOL data using validated instruments were included.

**Data Extraction and Synthesis:**

The review is reported according to the Preferred Reporting Items for Systematic Reviews and Meta-Analyses guideline. Meta-analysis was performed using random-effects models. All studies underwent qualitative synthesis and quality appraisal. Data were extracted and quality was assessed by 2 independent reviewers.

**Main Outcomes and Measures:**

Meta-analysis was carried out on studies that used the World Health Organization QOL–Short Form (WHOQOL-BREF; range, 0-100, with higher scores indicating better QOL) and the 12-Item Short Form Health Survey (SF-12; mean score, 50, with higher scores indicating better QOL) to assess the QOL among parents of preterm children. Qualitative analysis was performed to compare QOL of parents following preterm and full-term births, fathers vs mothers, and by degree of prematurity. Modifiable factors associated with QOL were identified by synthesizing evidence from longitudinal cohort studies and randomized clinical trials.

**Results:**

A total of 34 studies, with 6617 parents of preterm children and 8295 parents of full-term children, were included. Meta-analysis of WHOQOL-BREF data (10 studies, 1147 parents of preterm children) showed pooled mean domain scores ranged from 63.63 (95% CI, 54.00-73.35) in the environmental domain to 66.68 (95% CI, 59.77-73.60) in the psychological well-being domain. Systematic review found that lower parental QOL was evident during infant hospitalization with improvement over time. Meta-analysis of SF-12 data (5 studies, 3137 parents) revealed pooled mean physical and mental component summary scores of 47.22 (95% CI, 40.06-54.39) and 44.58 (95% CI, 39.01-50.16), respectively. High heterogeneity reflected variability of infant maturity, assessment timing, and sociodemographic factors. Parents of preterm infants reported lower QOL than parents of full-term children in the early postpartum period, but this gap narrowed over time. Mothers reported poorer QOL compared with fathers during the infant’s hospitalization, but the gap diminished by 4 to 6 months post partum. Key modifiable factors of QOL were parental psychological well-being, knowledge and empowerment, social and systemic support, and infant-related health burdens.

**Conclusions and Relevance:**

In this systematic review and meta-analysis, parental QOL was lowest during the premature infant’s hospitalization. A family-centered approach, with timely and tailored support from the neonatal intensive care unit through the postdischarge period, is essential to protect and promote parental well-being.

## Key Points

**Question:**

What is the quality of life (QOL) of parents of premature infants, and what factors are associated with it?

**Findings:**

In this systematic review and meta-analysis of 34 studies with 6617 parents of preterm infants and 8295 parents of full-term infants, parental QOL was lowest during neonatal intensive care unit hospitalization. Key modifiable factors associated with QOL included parental psychological distress, lack of preparedness, and inadequate social support; disparities between mothers and fathers were most pronounced during the immediate postpartum period but narrowed over time.

**Meaning:**

In this study, birth and hospitalization of a premature infant was associated with lower parental QOL, but a trajectory of recovery could be supported by targeted interventions for mothers and socioeconomically disadvantaged parents through family-centered care, psychological support, and sleep hygiene.

## Teaser

This systematic review and meta-analysis examines the quality of life among parents of preterm infants and identifies modifiable factors associated with parental quality of life in the postpartum period.

## Introduction

The parent-child relationship is a complex, interdependent dyad in which parental well-being is closely tied to the child’s health status.^1^ In addition, parental stress and mental health can significantly affect the child’s developmental outcomes.^2^ The chronic stress of the neonatal intensive care unit (NICU) experience, ongoing caregiving demands, and fear about their child’s future can compromise the quality of life (QOL) of parents of premature infants.

Premature infants, born before 37 weeks of gestation, represent 10% of live births globally.^3^ They are a diverse group, with varying biological needs. Extremely premature infants, born at or before 28 weeks of gestation, typically require prolonged hospitalization and ongoing medical support after discharge, with risks of long-term neurodevelopmental, growth, and respiratory complications.^4,5,6^

QOL is defined as an “individual’s perception of their position in life in the context of the culture and value systems in which they live and in relation to their goals, expectations, standards and concerns.”^7^ It is a holistic marker of well-being, encompassing physical, psychological, and social domains.^7^ Consequently, parental mental health has been identified as a research priority by a multinational consortium comprising clinicians, parents, and adults born preterm.^8^ While previous studies have examined parental mental health, a comprehensive synthesis focusing specifically on multidomain QOL and its modifiable determinants is lacking.

We undertook this systematic review and meta-analysis to assess the QOL of parents following preterm birth and identify modifiable factors associated with QOL, defined as factors that can be realistically targeted by health care or psychosocial interventions (eg, mitigating parenting stress, enhancing social support). The review question, structured using a modification of the participants, exposure, comparator, and outcome framework,^9^ is as follows: participants, parents; exposure, premature birth; comparator, parents of full-term (≥37 weeks of gestation) children, where available; and outcomes, QOL of parents and factors associated with it. We expect our review to assist health care practitioners in identifying parents of preterm infants who may be at risk of poorer QOL and to establish an evidence base to inform the development of targeted interventions and supportive policies to promote parental well-being.

## Methods

We report this review according to Preferred Reporting Items for Systematic Reviews and Meta-Analyses (PRISMA) reporting guideline.^10^ We registered the protocol with Prospective Register of Systematic Reviews on March 26, 2024 (CRD42024523517). eTable 1 in Supplement 1 highlights differences between the a priori PROSPERO protocol and the final review.

### Search Strategy

We devised the search strategy (eTable 2 in Supplement 1) in conjunction with a medical librarian. Search terms included following key words and related MeSH terms: *parents*, *father*, *mother*, *quality of life*, *health related quality of life*, *premature*, *caregiving burden*, *parental stress*, and selected QOL instruments (World Health Organization QOL–Short Form [WHOQOL-BREF], 8-Item Short Form Health Survey [SF-8], 12-Item Short Form Health Survey [SF-12], 36-Item Short Form Health Survey [SF-36], and PedsQL Family Impact Module [PedsQL FIM]). We searched PubMed, CINAHL, and Embase from inception to October 5, 2025, and searched the bibliographies of included articles. We used Covidence to manage the review process.

### Inclusion and Exclusion Criteria

We included cross-sectional studies, longitudinal cohort studies, and randomized clinical trials (RCTs) published in English that used validated tools to quantitatively assess QOL of parents with premature infants. Studies examining the association of QOL with psychological factors (eg, anxiety, depression, coping mechanisms, stress) and socioeconomic status were included. We excluded studies on QOL instrument validation as well as reviews and non–peer reviewed articles (eg, abstracts, preprints). eTable 3 in Supplement 1 provides detailed inclusion and exclusion criteria.

### Study Selection and Data Extraction

Two reviewers (S.A.S.E.Y. and Q.Z.X.L.) independently screened title and abstract, followed by full text for study eligibility. These reviewers used a standardized, piloted form to extract data (study design, participants’ demographics, QOL instruments and versions, assessment time points, quantitative results) and compared the data. We resolved discrepancies through team consensus. We contacted study authors for missing data or clarifications.

### Quality Appraisal of Included Studies

Two reviewers (S.A.S.E.Y. and Q.Z.X.L.) independently performed quality appraisal using the Newcastle-Ottawa Scale (NOS).^11^ We treated RCTs (preintervention data) and longitudinal cohorts (data from first time point) as cross-sectional studies. As the original NOS was designed for cohort and case-control studies, we used an adaptation for cross-sectional studies, which involved the removal of an item specific to longitudinal design (ie, adequacy of follow-up of cohorts).^12^ The NOS was selected as it provides a consistent framework to appraise the quality of such studies, with a focus on participant selection, comparability, and the ascertainment of the outcome of interest, allowing for comparability across studies.

### Statistical Analysis

All meta-analyses were conducted using R software version 4.3.1 (R Project for Statistical Computing) with the meta package version 7.0-0. A *P *less than .05 was considered statistically significant. We fitted random-effects models using the restricted maximum likelihood estimator to compute pooled estimates and quantify heterogeneity.

We conducted separate meta-analyses for each QOL instrument to ensure conceptual consistency, as operational definitions of QOL domains vary across tools. We did not pool scores from different instruments (eg, WHOQOL-BREF vs SF-12) using standardized mean differences, as they measure distinct constructs.

For meta-analysis, which aimed to estimate the baseline QOL, we extracted data from the preintervention time point in RCTs and the first available time point in longitudinal cohort studies to ensure all data represented an initial state prior to specific interventions or significant temporal changes. Data from all time points were considered for the qualitative synthesis.

We conducted meta-analysis for outcomes for which data were available from a minimum of 4 independent studies using the same QOL tool. This threshold ensured a reliable estimation of the pooled effect size and between-study heterogeneity, which is unstable with fewer studies.^13^ We performed subgroup analysis on full-term vs preterm parental QOL.

Forest plots were constructed using means and SDs of QOL scores. Medians and IQRs were converted to means and SDs using the method recommended by Hozo et al.^14^

Statistical heterogeneity was assessed with several complementary measures.^13^ The *I*^2^ statistic quantified proportion of total variability in effect estimates that is due to heterogeneity rather than chance. The tau-squared (τ^2^) and its SD directly estimated the magnitude of heterogeneity from between-study variance. We computed 95% prediction intervals to describe the expected range of true effects across similar future studies.

We conducted qualitative synthesis guided by Popay et al.^15^ First, extracted data were systematically organized and summarized in Excel version 2510 (Microsoft Corp). Next, data were examined to identify relationships and patterns. This involved a thematic analysis of the reported modifiable factors and experiences related to QOL. Finally, the robustness of the synthesis was strengthened through iterative comparison of themes across studies and critical reflection on how individual study design and contexts might have influenced the findings.

To strengthen causal inference, we identified modifiable factors associated with QOL and potential interventions with evidence from longitudinal cohort studies and RCTs. These study designs offer better temporal link between the factors and QOL outcomes than cross-sectional studies.

## Results

**Figure 1.**
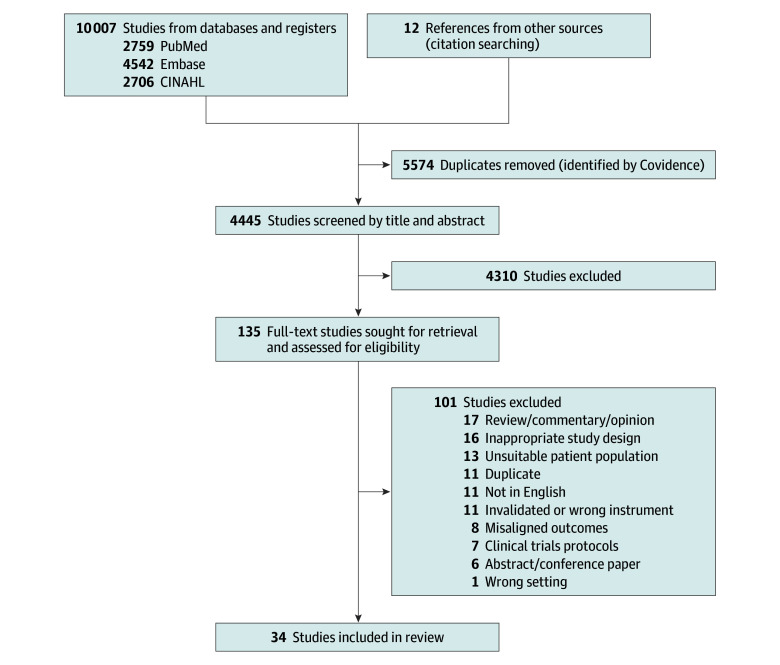
Study Flowchart

We identified 10 007 articles from database search and another 12 through hand-search. After deduplication and screening of titles and abstracts, we retrieved 135 full-text articles. We included 34 studies^16,17,18,19,20,21,22,23,24,25,26,27,28,29,30,31,32,33,34,35,36,37,38,39,40,41,42,43,44,45,46,47,48,49^ involving 6617 parents of preterm children (4546 mothers, 303 fathers, and 1768 unspecified parents) and 8295 parents of term children (3593 mothers, 1106 fathers, and 3596 unspecified parents). Figure 1 shows the PRISMA flow diagram.

eTable 4 in Supplement 1 summarizes the main characteristics of included studies.^16,17,18,19,20,21,22,23,24,25,26,27,28,29,30,31,32,33,34,35,36,37,38,39,40,41,42,43,44,45,46,47,48,49^ The children represented following gestational age distribution: 28 or fewer weeks, 20%; 29 to 32 weeks, 36%; 33 to 36 weeks, 15%; unspecified, 29%. In most studies (24 [71%]),^16,17,18,19,20,21,22,23,24,29,30,34,35,36,37,38,39,40,41,42,44,45,46,47^ QOL was assessed within the first year post partum. Twenty-one studies^24,25,26,27,28,29,30,31,32,33,35,37,39,40,42,43,44,45,47,48,49^ were conducted in high-income countries, 5 studies^17,21,22,34,46^ in upper-middle-income countries, and 8 studies^16,18,19,20,23,36,38,41^ in lower-middle-income countries as per classification by the Organisation for Economic Co-operation and Development.^50^ There were 18 cross-sectional studies,^22,23,24,25,28,29,30,31,32,33,35,36,40,42,43,47,48,49^ 11 longitudinal cohort studies^16,17,19,21,26,27,37,39,41,45,46^ and 5 RCTs.^18,20,34,38,44^ There was no mixed method study. In 3 studies,^21,22,25^ median and IQR values were converted to mean and SD values for meta-analysis. The main QOL instruments used are described in the eMethods in Supplement 1.

### Quality of Studies

The quality of 33 of 34 studies (97%) was satisfactory to very good (eTable 5 in Supplement 1). Interrater reliability between the 2 reviewers was 91%.

### Quantitative Synthesis

#### Total and Domain QOL Scores in WHOQOL-BREF and SF-12

**Figure 2.**
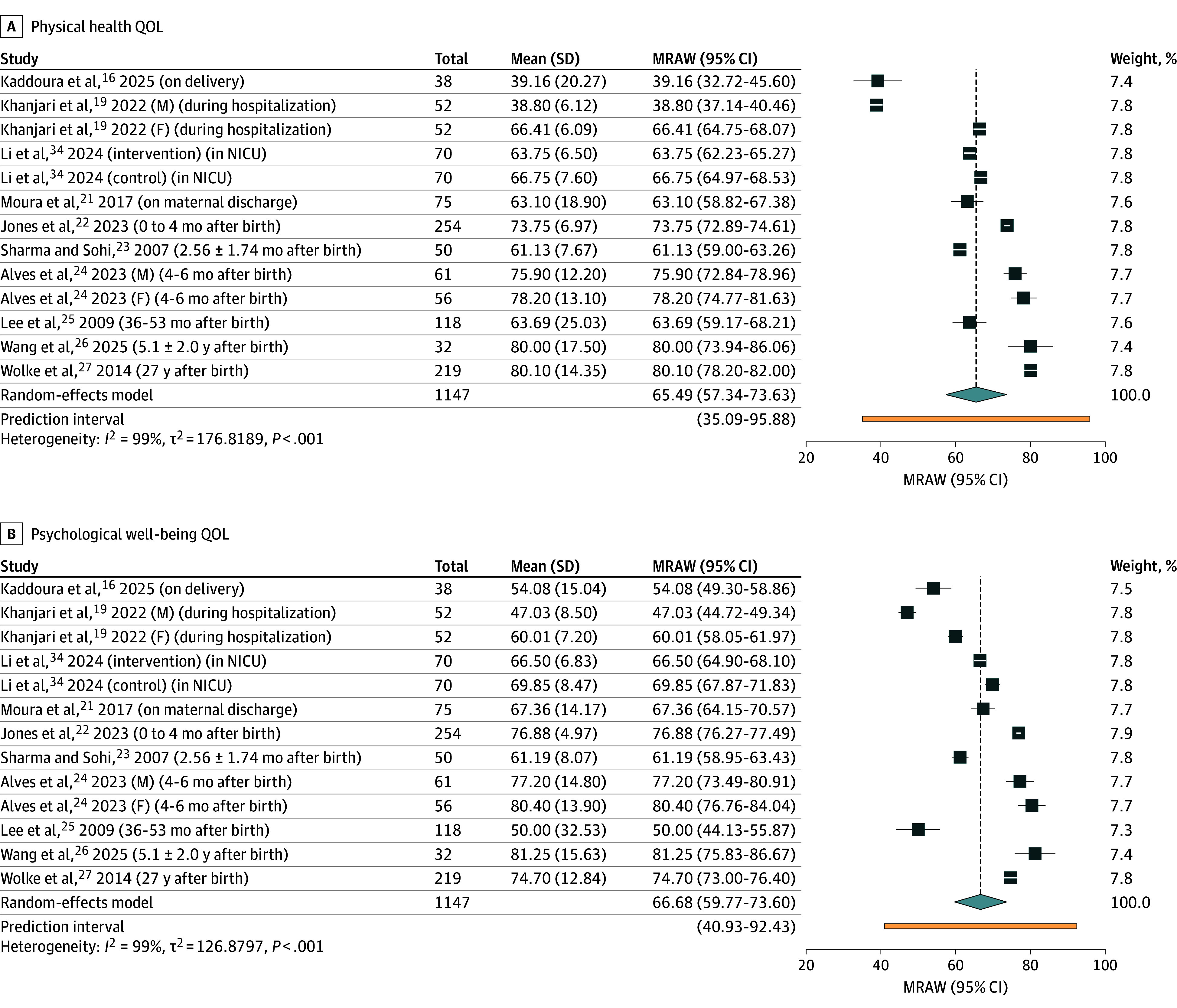
Forest Plots of Studies With the World Health Organization Quality of Life (QOL)–Short Form Physical Health and Psychological Well-Being Domains F indicates father; M, mother; MRAW, meta-analysis of raw means; NICU, neonatal intensive care unit.

**Figure 3.**
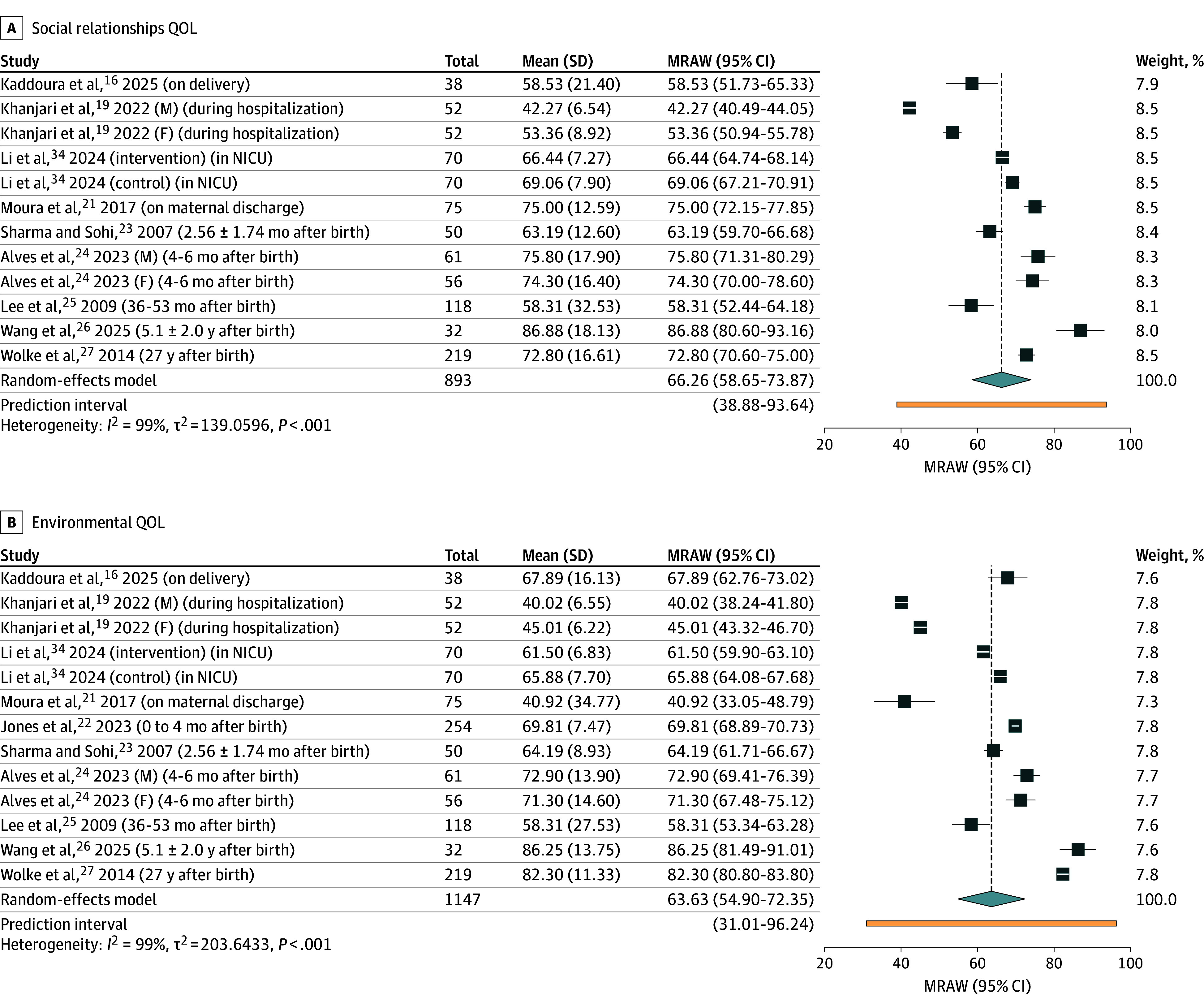
Forest Plots of Studies With the World Health Organization Quality of Life (QOL)–Short Form Social Relationships and Environmental Domains F indicates father; M, mother; MRAW, meta-analysis of raw means; NICU, neonatal intensive care unit.

Thirteen studies (with 1447 parents) reported QOL with WHOQOL-BREF.^16,17,18,19,20,21,22,23,24,25,26,27,34^ Ten studies (with 1147 parents) underwent meta-analysis,^16,19,21,22,23,24,25,26,27,34^ while 3 studies were excluded due to inadequate reporting of data.^17,18,20^ Across 4 domains, parental QOL was lowest during the immediate postpartum period and while infant was hospitalized in the NICU, with scores increasing over time. Figures 2 and 3 presents forest plots of WHOQOL-BREF domain scores. The environmental domain had the lowest pooled mean score (63.63; 95% CI, 54.90-72.35) (Figure 3), followed by physical health (65.49; 95% CI, 57.34-73.63) (Figure 2), social relationships (66.26; 95% CI, 58.65-73.87) (Figure 3), and psychological health (66.68; 95% CI, 59.77-73.60) (Figure 2). Heterogeneity was substantial (*I^2^* = 99%; *P* < .01) with wide prediction intervals and high τ^2^ values in all subdomains, reflecting variability in study populations, timing of assessment, and contextual factors.

**Figure 4.**
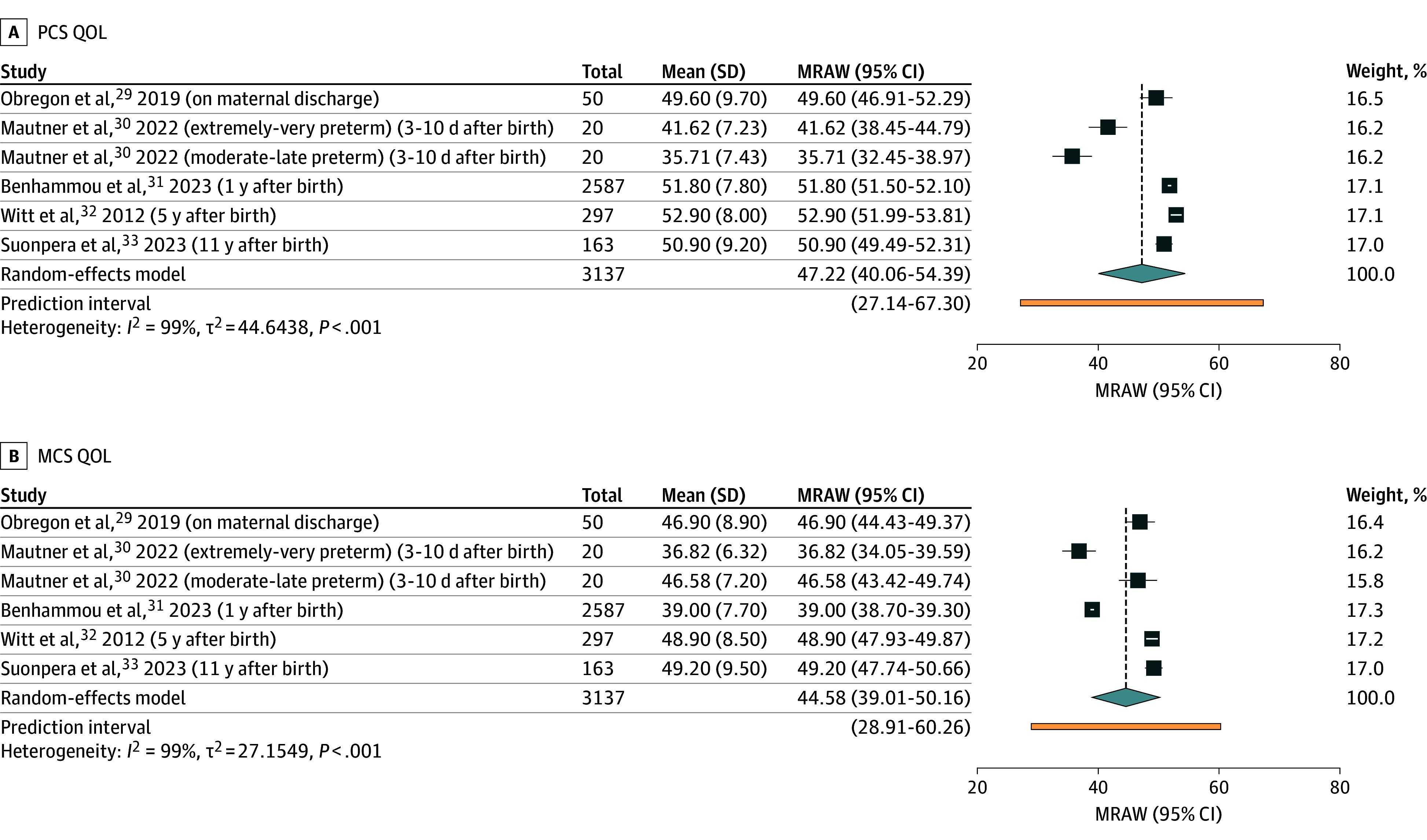
Forest Plots of Studies Using 12-Item Short Form Health Survey MCS indicates mental component summary; MRAW, meta-analysis of raw means; PCS, physical component summary; QOL, quality of life.

Figure 4 presents forest plots of parental QOL using SF-12 from 5 studies (with 3137 parents).^29,30,31,32,33^ The pooled mean physical component summary (PCS) score was 47.22 (95% CI, 40.06-54.39), and the pooled mean mental component summary (MCS) score was 44.58 (95% CI, 39.01-50.16). The timing of QOL assessment varied across studies, ranging from 3 to 10 days post partum^30^ to 11 years after birth,^33^ contributing to substantial heterogeneity (*I^2^* = 96% for PCS and *I^2^* = 99% for MCS; *P* < .01) with wide prediction interval and high τ^2^ values.

**Figure 5.**
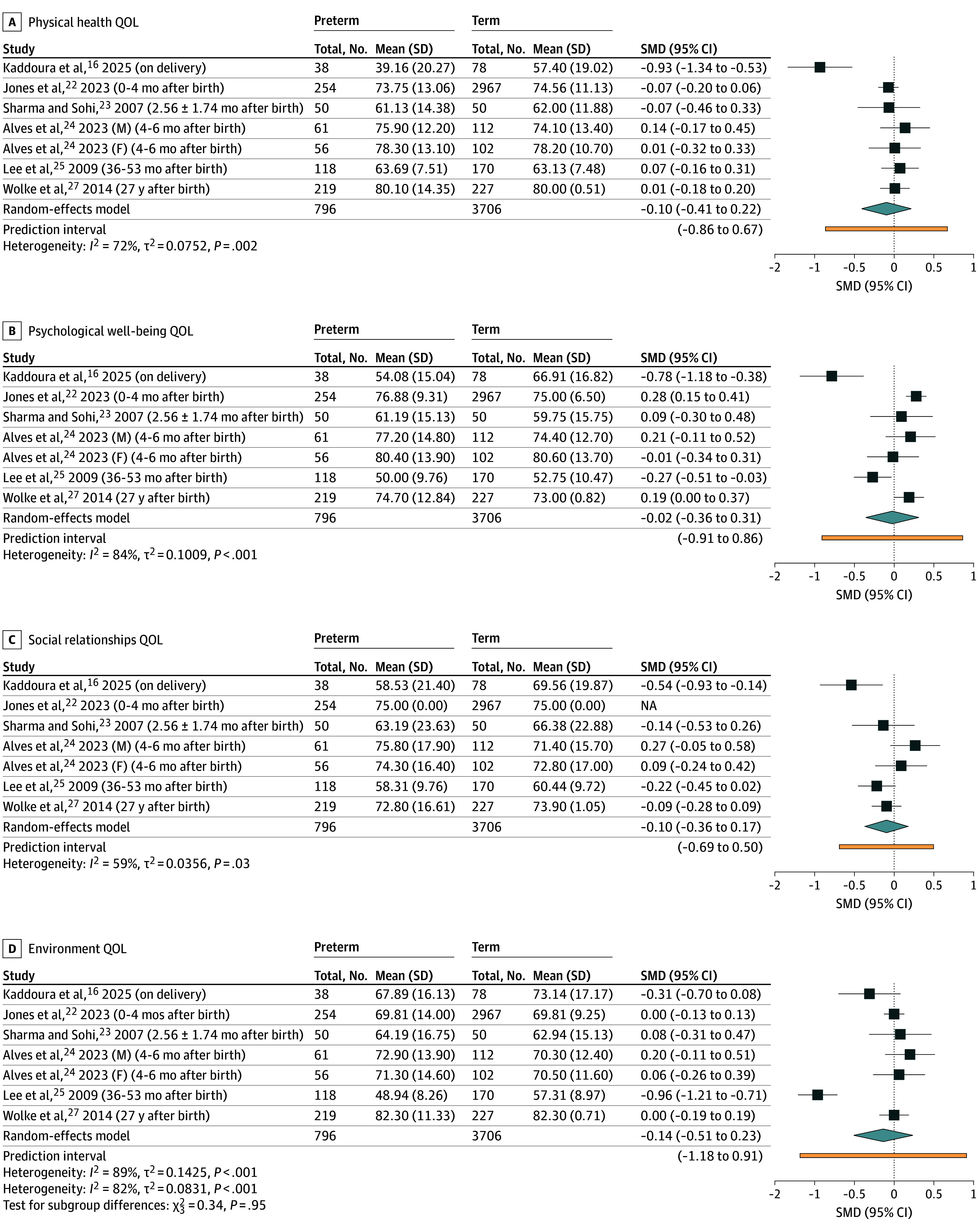
Forest Plot Comparing World Health Organization Quality of Life (QOL)–Short Form Scores Between Parents of Preterm and Full-Term Infants SMD indicates standardized mean difference.

We conducted a subgroup meta-analysis on 6 WHOQOL-BREF studies reporting experiences of parents with preterm and full-term children (796 parents of preterm children and 3706 parents of full-term children) (Figure 5).^16,22,23,24,25,27^ The timing of QOL assessment ranged widely, from immediately after delivery^16^ to 27 years after birth.^27^ Across all 4 domains—physical health, psychological well-being, social relationships, and environment—there were no significant differences in QOL scores. Heterogeneity varied across domains, from moderate in the social relationships domain (*I^2^* = 59%) to high in the environmental domain (*I^2^* = 89%).

### Qualitative Synthesis

#### Parents of Preterm and Full-Term Infants

Six studies^16,22,23,24,25,27^ using WHOQOL-BREF conducted from within the first year post partum^22,23,24^ to 27 years of child’s age^27^ did not show differences in QOL scores with parents of full-term infants, similar to findings from the meta-analysis. However, in Lebanon during a period of compounded social and economic crises, mothers of preterm infants reported significantly lower QOL across physical, psychological, and social domains immediately after birth compared with mothers of full-term infants.^16^ Their physical and psychosocial domain scores improved at 4 to 6 months post partum, narrowing the gap with those of mothers of full-term infants.^16^

In contrast, cross-sectional studies measuring health-related QOL using SF-12 showed heterogeneous outcomes.^30,32,33^ Compared with full-term control participants, mothers of preterm infants had lower MCS but higher PCS scores in the immediate postpartum period in Austria,^30^ lower MCS and PCS when children were aged 5 years in the United States,^32^ and comparable PCS and MCS scores when children were aged 11 years in England.^33^ A longitudinal study using the SF-36 in Norway demonstrated no difference in QOL between parents of preterm and full-term infants until 12 months post partum.^39^

McAndrew et al,^40^ using PedsQL FIM, highlighted a dynamic trajectory; parents of extremely preterm infants had low QOL during NICU admissions but demonstrated the highest degree of improvement in emotional functioning and family relationships 3 months after discharge compared with parents of full-term infants. Petersen and Quinlivan,^46^ who exclusively evaluated paternal QOL, found that fathers of premature infants compared with fathers of full-term infants had higher rates of anxiety and lower QOL at 6 weeks after birth, despite having similar scores during the antenatal period.

#### Degree of Prematurity

Three studies assessed parental QOL stratified by degree of prematurity.^30,40,45^ The degree of prematurity was not linearly associated with QOL. Although parents of extremely preterm infants (≤28 weeks of gestation) reported lower QOL during the NICU stay, their QOL tended to improve over time, particularly after discharge.^40^ Mothers of preterm infants had significantly lower psychological well-being compared with those with near-term or full-term infants, with improvement in overall QOL over the first 3 weeks post partum.^45^ However, another study found that physical health–related QOL of mothers of moderate to late preterm infants (32-37 weeks of gestation) was lower than that of mothers of very preterm infants (<32 weeks of gestation), and they experienced significant physical strain and psychological distress, particularly during the early postpartum period.^30^

#### Differences Between Fathers and Mothers

Synthesis of 5 studies^19,24,39,41,47^ revealed a temporal pattern in the comparative experiences of mothers and fathers. When QOL assessments were conducted early (ie, during NICU admission or within the first month post partum), mothers consistently reported poorer QOL.^19,37,41^ However, this disparity appeared to diminish over time, with no differences in QOL when assessed at 4 to 6 months^24^ and at 2, 6, and 12 months.^39^ Interestingly, another study reported better maternal QOL in time limitations and emotional domains up to 2 years after discharge.^47^

#### Factors Associated With QOL

Synthesis of longitudinal and RCTs identified 4 key modifiable factors associated with parental QOL. The first was parental psychological and emotional well-being. Elevated levels of perceived stress, anxiety, depression, and posttraumatic stress symptoms—in mothers and fathers—during the NICU stay and postdischarge period were consistently associated with poor QOL.^21,37,41,46^ Likewise, targeted interventions, such as spiritual self-care training and web-based diaries, were associated with improved QOL.^20,34^

Second, deficits in knowledge, preparedness, and empowerment were significantly associated with impaired QOL. Lack of confidence in caregiving skills and feeling unprepared for discharge were major stressors. Structured educational programs, with family-centered care approaches and discharge preparation, were associated with improved QOL by building parental competence and self-efficacy.^18,19^

Third, lower level of social and systemic support, including perceived support from partners, family, and friends, were associated with poorer QOL.^16,37^ Conversely, enhanced, empathetic communication from staff and participation in structured empowerment programs like Creating Opportunities for Parenting Experience (COPE) were associated with better QOL and well-being.^41,44^ Peer support also improved parental mental health and social functioning.^38^

Finally, infant-related health factors and associated practical burdens represented a key target for supportive interventions. Stress related to the infant’s behavior and growth and the burden of managing chronic conditions were negatively associated with QOL.^21,37,41^ Financial strain and sleep disruption were also significant and actionable stressors that were mitigated through targeted support.^39,41^

## Discussion

In this systematic review and meta-analysis of 34 studies, we found that QOL for parents of premature children was lowest during their infant’s hospitalization in the NICU, with a trajectory of recovery over time. Meta-analysis of 6 studies using WHOQOL-BREF, which measures generic QOL, from the perinatal period to adulthood, found no difference in QOL between parents of full-term and preterm infants. However, the lack of statistical difference in meta-analysis of WHOQOL-BREF should be interpreted with caution given that results showed significant heterogeneity and wide confidence intervals. SF-based studies, measuring health-related QOL, reported psychological vulnerability in parents of preterm infants during early childhood. Notably, the degree of prematurity was not linearly associated with QOL outcomes.

Mothers reported lower QOL scores than fathers, especially during the early postpartum period, with disparities diminishing over time. This finding is consistent with greater maternal vulnerability after childbirth. Emerging evidence also highlighted the distinct roles and coping mechanisms employed by fathers.^51^ Fathers who reported higher subjective birth-related stress were more likely to experience depressive symptoms 6 months post partum.^52^ However, current NICU parent support programs are often focused on the mother,^53^ underestimating the unique needs of fathers. These findings underline the importance of gender-sensitive support—providing mothers with emotional and physical help after birth and helping fathers participate more in caregiving with appropriate psychosocial and emotional support.

This study identified several modifiable factors associated with QOL. Psychological distress—stress, anxiety, depression, and posttraumatic symptoms—was consistently associated with poorer QOL. Interventions such as spiritual self-care training, web-based diaries, and peer support programs demonstrated efficacy in improving psychological well-being. Deficits in caregiving confidence and discharge preparedness also impaired QOL, while structured educational programs and empowerment initiatives improved parental competence and self-efficacy. Social support, both informal and institutional, emerged as a critical buffer against stress, with enhanced communication from health care staff and participation in programs like COPE associated with better outcomes. Infant-related health burdens, financial strain, and sleep disruption were additional modifiable stressors that warrant targeted intervention.

We recommend that clinical pathways for families of preterm infants incorporate brief, validated QOL screenings, such as the SF-12, and mental health screenings at key transition points (eg, NICU admission, discharge, and 3 to 6 months after discharge) with clear referral pathways to psychological and social services. As the parent-child dyadic relationship is reciprocal, a positive change in parental well-being is likely to have a lasting impact on the outcome of their premature children.

Future research should systematically recruit fathers and examine dyadic coping processes to inform gender-sensitive interventions. There is a pressing need to evaluate QOL from lower-income countries, which were not represented in this review. Longitudinal studies are needed to map the evolving trajectories of QOL among parents across infancy, childhood, and into adolescence. Additionally, RCTs evaluating integrated, culturally adaptable support models to improve QOL can provide further evidence for effective interventions to promote family well-being.

### Limitations

We would like to highlight several limitations. The heterogeneity in the meta-analyses is the result of diverse infant characteristics, settings, timing of QOL assessments, and study methods. All studies reported QOL only after the child’s birth, except for a single study^46^ reporting paternal QOL at the prenatal and 6 weeks postnatal period. Consequently, the observed impact of preterm birth after delivery could be confounded by preexisting parental states. We could not determine whether fathers and mothers were from the same families, limiting our ability to discern the association of family dynamics with QOL. In the subgroup analysis comparing parents of preterm and full-term infants, the health status of the full-term–born control group was largely unreported, except for one study.^24^ There were missing QOL values in some of the domains of reported studies, raising the possibility of reporting bias. Only studies published in English were included. Specific versions of the QOL instruments used were often not reported in the primary studies. Although the core domains of these instruments are generally consistent, this lack of reporting introduced a potential source of unmeasured heterogeneity into our synthesis. There were no studies from low-income countries, although some of these countries have among the highest percentages of premature births.^3^ Fathers were underrepresented in this review.

## Conclusions

In this systematic review and meta-analysis, parents of premature infants experienced the lowest QOL during their children’s hospitalization. The modifiable factors associated with QOL that we identified, including psychological well-being, parental education, and social support, could form the basis for interventions to support the QOL of parents.

## Notes

Supplement 1. **eMethods.** Main Quality of Life Tools Used **eTable 1.** Differences Between PROSPERO Protocol and the Final Study **eTable 2.** Search Strategy **eTable 3.** Inclusion and Exclusion Criteria **eTable 4.** Characteristics and Quality of Life Outcomes of Included Studies, Grouped by Instrument **eTable 5.** Newcastle- Ottawa Scale for Cross Sectional Studies
eReferences.

Supplement 2.
Data Sharing Statement
