# Supplementary material for: Quality of Life of Parents of Premature Infants: A Systematic Review and Meta-Analysis
Source: JAMA Netw Open. 2026 Jan 14;9(1):e2553712. doi: 10.1001/jamanetworkopen.2025.53712 (PMC12805452; doi:10.1001/jamanetworkopen.2025.53712)
Supplement: Supplement 1. — eMethods. Main Quality of Life Tools Used eTable 1. Differences Between PROSPERO Protocol and the Final Study eTable 2. Search Strategy eTable 3. Inclusion and Exclusion Criteria eTable 4. Characteristics and Quality of Life Outcomes of Included Studies, Grouped by Instrument eTable 5. Newcastle- Ottawa Scale for Cross Sectional Studies eReferences. [file jamanetwopen-e2553712-s001.pdf]

Supplemental Online Content

Yip SASE, Lim QZ, Kong G, Amin Z, Ng YPM. Quality of life of parents of premature infants: a systematic review and meta-analysis. *JAMA Netw Open.* 2026;9(1):e2553712. doi:10.1001/jamanetworkopen.2025.53712

eMethods. Main Quality of Life Tools Used

eTable 1. Differences Between PROSPERO Protocol and the Final Study

eTable 2. Search Strategy

eTable 3. Inclusion and Exclusion Criteria

eTable 4. Characteristics and Quality of Life Outcomes of Included Studies, Grouped by Instrument

eTable 5. Newcastle- Ottawa Scale for Cross Sectional Studies

eReferences.

This supplemental material has been provided by the authors to give readers additional information about their work.

© 2026 Yip SASE et al. *JAMA Network Open*.

eMethods. Main Quality of Life Tools Used

1. WHOQOL-BREF

The WHOQOL-BREF is a condensed 26-item instrument derived from the comprehensive WHOQOL-100, designed to evaluate quality of life (QOL) across four core domains: *Physical Health* (7 items), *Psychological Health* (6 items), *Social Relationships* (3 items), and *Environmental Health* (8 items).[1] Two standalone items assess overall QOL and general health. Responses are recorded on a five-point Likert scale. Raw domain scores are standardized using a two-step process:

- **Step 1:** Domain means are scaled to a 4–20 range for compatibility with the WHOQOL-100.
- **Step 2:** Scores are linearly converted to a 0–100 scale using the formula:

Transformed Score = ((Raw Domain Score - 4) / (20 - 4)) * 100

Higher scores reflect better perceived QOL.[2]

2. Short Form Health Surveys (SF-36 and SF-12)

- **SF-36:** This 36-item survey assesses health-related quality of life (HRQoL) through eight subdomains: *Physical Functioning*, *Role Limitations-Physical*, *Bodily Pain*, *General Health*, *Vitality*, *Social Functioning*, *Role Limitations-Emotional*, and *Mental Health*. Items use variable response levels (4–21 points), with higher scores indicating better health status despite impairments.[3]
- **SF-12:** A streamlined version of the SF-36, this 12-item tool replicates the Physical Component Summary (PCS) and Mental Component Summary (MCS) scales. The PCS integrates *Physical Functioning*, *Role-Physical*, *Bodily Pain*, and *General Health*, while the MCS includes *Vitality*, *Social Functioning*, *Role-Emotional*, and *Mental Health*. Validated in U.S. populations, the SF-12 accounts for ~85% of SF-36 variance and reduces completion time to <2 minutes.[4] The population norm score of MCS and PCS is 50.

© 2026 Yip SASE et al. *JAMA Network Open*.

3. PedsQL Family Impact Module (FIM 4.0)

The PedsQL FIM 4.0 is a 36-item parent-report questionnaire evaluating the impact of pediatric chronic health conditions on family functioning.[5] It measures eight domains:

- **Parent-Specific Domains:** *Physical Functioning*, *Emotional Functioning*, *Social Functioning*, *Cognitive Functioning*, *Communication*, and *Worry*.
- **Family-Specific Domains:** *Daily Activities* and *Family Relationships*.
  Three composite scores are generated: *Total Score* (all items), *Physical Health Summary* (physical functioning), and *Psychosocial Health Summary* (emotional, social, and cognitive functioning). Responses use a 5-point frequency scale (0 = *never* to 4 = *almost always*), reverse-scored and converted to a 0–100 scale, where higher scores denote better family HRQoL.[5]

© 2026 Yip SASE et al. *JAMA Network Open*.

eTable 1. Differences Between PROSPERO Protocol and the Final Study

	PROSPERO Protocol	Final Study
Database search	PubMed, Web of Science, CINAHL, PsycINFO, Embase, CNKI	PubMed, CINAHL, Embase
Search date	March 2024	25th April 2025<br>Updated search 4th October 2025
Sub-group analysis	Different gestational age (e.g., very preterm (29-32 weeks) and extreme preterm (less than 28 weeks)), age when study was performed (i.e. early childhood, late childhood, teenage years), geographical areas, respondents (mother or father) with available data.	We could not perform quantitative synthesis (i.e., meta-analysis) based on gestational age, age when study was performed, geographical areas, gender (mother or father) due to inadequate number of suitable studies. We performed qualitative synthesis instead.

© 2026 Yip SASE et al. *JAMA Network Open*.

eTable 2. Search Strategy

PubMed

1.	"Parents"[MeSH]
2.	Parent*[Title/Abstract] OR mother*[Title/Abstract] OR father*[Title/Abstract] OR parental[Title/Abstract] OR Caregiver*[Title/Abstract] OR maternal[Title/Abstract]
3.	#1 or #2
4.	"Premature Birth"[MeSH] OR "Infant, Premature"[MeSH]
5.	Prematur*[Title/Abstract] OR preterm[Title/Abstract] OR Low birth weight[Title/Abstract]
6.	#4 or #5
7.	"Quality of Life"[MeSH]
8.	Quality of Life[Title/Abstract] OR Life Quality [Title/Abstract] OR QOL[Title/Abstract] OR HRQOL[Title/Abstract] OR Health related quality of life [Title/Abstract] OR Life satisfaction[Title/Abstract] OR Wellbeing[Title/Abstract] OR Parental stress[Title/Abstract] OR Caregiving burden[Title/Abstract]
9.	SF-8[Title/Abstract] OR SF-12[Title/Abstract] OR SF-36[Title/Abstract] OR "Short Form 8"[Title/Abstract] OR "Short Form 12"[Title/Abstract] OR "Short Form 36"[Title/Abstract] OR WHOQOL[Title/Abstract] OR "World Health Organization Quality of Life"[Title/Abstract] OR PedsQL "Family Impact Module"[Title/Abstract]
10.	#7 OR #8 OR #9
11.	#3 AND #6 AND #10

© 2026 Yip SASE et al. *JAMA Network Open*.

Embase

1	exp parent/
2	(parent* or mother* or father* or parental or caregiver* or maternal).ab,ti.
3	exp prematurity/
4	(prematur* or preterm or pre-term or low birth weight).ab,ti.
5	exp "quality of life"/
6	(quality of life or life quality or QOL or HRQOL or health related quality of life or life satisfaction or wellbeing or parental stress or caregiving burden).ab,ti.
7	(sf-8 or sf-12 or sf-36 or short form 8 or short form 12 or short form 36 or WHOQOL or world health organization quality of life or pedsql family impact module).ab,ti.
8	1 or 2
9	3 or 4
10	5 or 6 or 7
11	exp premature labor/
12	9 or 11
13	8 and 10 and 12

© 2026 Yip SASE et al. *JAMA Network Open*.

CINAHL

1.	(MH "Parents+")
2.	TI (Parent* OR mother* OR father* OR parental OR Caregiver* OR maternal) OR AB (Parent* OR mother* OR father* OR parental OR Caregiver* OR maternal)
3.	S1 OR S2
4.	(MH "Childbirth, Premature")
5.	(MH "Infant, Premature")
6.	S4 OR S5
7.	TI (Prematur* OR preterm OR pre-term OR Low birth weight) OR AB (Prematur* OR preterm OR pre-term OR Low birth weight)
8.	S6 OR S7
9.	(MH "Quality of Life+")
10.	TI (Quality of Life OR Life Quality OR QOL OR HRQOL OR Health related quality of life OR Life satisfaction OR Wellbeing OR Parental stress OR Caregiving burden) OR AB (Quality of Life OR Life Quality OR QOL OR HRQOL OR Health related quality of life OR Life satisfaction OR Wellbeing OR Parental stress OR Caregiving burden)
11.	(SF-8 OR SF-12 OR SF-36 OR "Short Form 8" OR "Short Form 12" OR "Short Form 36" OR WHOQOL OR "World Health Organization Quality of Life" OR PedsQL "Family Impact Module") AND (SF-8 OR SF-12 OR SF-36 OR "Short Form 8" OR "Short Form 12" OR "Short Form 36" OR WHOQOL OR "World Health Organization Quality of Life" OR PedsQL "Family Impact Module")
12.	S9 OR S10 OR S11
13.	S3 AND S8 AND S12

© 2026 Yip SASE et al. *JAMA Network Open*.

eTable 3. Inclusion and Exclusion Criteria

	Inclusion Criteria	Exclusion Criteria
Population	Studies reporting quality of life of parents of children born premature	Studies reporting quality of life of premature infants or other caregivers or family members (e.g., siblings or grandparents)
Exposure	Prematurity (<37 weeks of gestation) of any degree.	Sick newborns who are not born premature
Instrument	Validated QOL instruments	Unvalidated or those undergoing validation process
Outcomes	Quality of life of parents of children born premature from their own perspectives	Studies of quality of life parents reported by others such as healthcare professionals
Nature of publications	Quantitative studies<br>Mixed method studies where quantitative data are available	Qualitative studies<br>Validation studies of instruments<br>Reviews, opinions, and consensus statements
Peer review process	Studies that underwent peer review process and published in journals	Publications that are not subject to a formal peer review process, such as conference reports, abstracts, and preprints
Language	English language	Publication in non-English languages

© 2026 Yip SASE et al. *JAMA Network Open*.

eTable 4. Characteristics and Quality of Life Outcomes of Included Studies, Grouped by Instrument

Source, study period, country, version used	Study design, comparator group	No. of participants, gender	Timing of assessment	Preterm infant (gestational age, birth weight), mean±SD/median (IQR)	Total score, mean± SD	Mean±SD/median (IQR)			
						Physical health	Psychological health	Social relationships	Environment
Studies utilizing WHOQOL-BREF (13)									
Kaddoura et al,[6] 2025, September 2021-December 2023, Lebanon WHOBREF Arabic version	Cohort study, yes	T1: 38 (preterm), 78 (term)<br>T2: 24 (preterm), 45 (term), mothers	T1: Upon delivery<br>T2: 4 to 6 mo postpartum	T1: 34.79 ± 1.60 wk, 2332.87 ± 429.74g<br>T2: 34.79 ± 1.60 wk, 2332.87 ± 429.74g	NA	T1: 39.16 ± 20.27<br>T2: 55.92 ± 23.76	T1: 54.08 ± 15.04<br>T2: 62.08 ± 17.82	T1: 58.53 ± 21.40<br>T2: 55.12 ± 26.78	T1: 67.89 ± 16.13<br>T2: 63.96 ± 20.27
Ong et al,[7] 2022, December 2015-June 2016, Malaysia WHOBREF Malay version	Cohort study, no	180, mothers	Within 48h of infant admission to 2 wk later	30.5 wk, <1500g	66.75 ± 18.25[a]	62.00 ± 5.50[a]	65.25 ± 5.56[a]	70.85 ± 5.06[a]	66.50 ± 5.31[a]
Neyestani et al,[8] 2017 (control), December 2015-May 2016, Iran	RCT, no	30, mothers	T1: First 48h of mother's attendance at infant's bedside<br>T2: 1-2 d before discharge<br>T3: 4 wk after discharge	32.7 ± 1.7 wk, 2076.1 ± 567g	T1: 69.7 ± 11.1	T1: 45.35 ± 26.88[a]	T1: 52.07 ± 18.13[a]	T1: 48.35 ± 10.63[a]	T1: 45.63 ± 21.88[a]
Neyestani et al,[8] 2017 (intervention), December 2015-May 2016, Iran	RCT, no	30, mothers	T1: First 48h of mother's attendance at infant's bedside<br>T2: 1-2 d before discharge<br>T3: 4 wk after discharge	32.1 ± 1.8 wk, 1941.1 ± 580g	T1: 72.0 ± 12.8	T1: 47.14 ± 30.00[a]	T1: 54.17 ± 19.38[a]	T1: 53.33 ± 13.13[a]	T1: 47.50 ± 25.00[a]

© 2026 Yip SASE et al. *JAMA Network Open*.

Khanjari et al,[9] 2022 (mothers), September - December 2020, Iran WHOBREF standardized Iran version	Cohort study, no	52, mothers	T1: During hospitalisatio n T2: 1 mo after parental education	30-36 wk, 1560.38 ± 326.797g	T1: 43.01 ± 3.99	T1: 38.80 ± 6.12	T1: 47.03 ± 8.50	T1: 42.27 ± 6.54	T1: 40.02 ± 6.55
Khanjari et al,[9] 2022 (fathers), September - December 2020, Iran WHOBREF standardized Iran version	Cohort study, no	52, fathers	T1: During hospitalisatio nT2: 1 mo after parental education	30-36 wk, 1560.38 ± 326.797g	T1: 56.98 ± 4.07	T1: 66.41 ± 6.09	T1: 60.01 ± 7.20	T1: 53.36 ± 8.92	T1: 45.01 ± 6.22
Sekhavatpour et al,[10] 2020 (control), 2012-2013, Iran WHOBREF Iranian version	RCT, no	30, mothers	T1: 5th day of infant hospitalisatio nT2: After intervention T3: 2 wk after intervention	32.2 ± 0.4 wk, NA	T1: 82.9 ± 1.8	NA	NA	NA	NA
Sekhavatpour et al,[10] 2020 (intervention), 2012-2013, Iran WHOBREF Iranian version	RCT, no	30, mothers	T1: 5th day of infant hospitalizatio nT2: After intervention T3: 2 wk after intervention	31.2 ± 2.0 wk, NA	T1: 84.2 ± 1.8	NA	NA	NA	NA
Li et al,[11] 2024 (control), May-December 2022, China WHOBREF Chinese version	RCT, no	70, unspecified	T1: in NICU, before intervention T2: Immediately after intervention T3: 1 mo after intervention	34.33 ± 2.32 wk, 2340 ± 540g	T1: 59.43 ± 8.30	T1: 66.75 ± 7.60[a]	T1: 69.85 ± 8.47[a]	T1: 69.06 ± 7.90[a]	T1: 65.88 ± 7.70[a]
Li et al,[11] 2024 (intervention), May-December 2022, China WHOBREF Chinese version	RCT, no	70, unspecified	T1: in NICU, before intervention T2: Immediately after intervention	34 ± 1.55 wk, 2210 ± 440g	T1: 57.31 ± 6.71	T1: 63.75 ± 6.50[a]	T1: 66.5 ± 6.83[a]	T1: 66.44 ± 7.27[a]	T1: 61.50 ± 6.83[a]

© 2026 Yip SASE et al. *JAMA Network Open*.

			T3: 1 mo after intervention						
Moura et al,[12] 2017, November 2009-June 2011, Brazil WHOBREF translated Brazilian version	Cohort study, no	T0: 75 T1: 63 T2: 44 T3: 47 T4: 32, mothers	T0: Upon maternal discharge T1: 6 mo post discharge T2: 12 mo post discharge T3: 24 mo post discharge T4: 36 mo post discharge	T0: 32.2 (28.3-34.0) wk, 1180 (585-1490) g T1: 31.4 (26.0-34.0) wk, 1170 (585-1470) g T2: 31 .4 (26.0-34.0) wk, 1145 (585-1470) g T3: 31.4 (26.0-34.0) wk, 1135 (585-1490) g T4: 31.4 (26.0-34.0) wk, 1077 (760-1470) g	NA	T0: 64.29 (50.00-75.00) T1: 75.00 (61.61-82.14) T2: 75.00 (64.29-82.14) T3: 67.86 (57.14-78.54) T4: 67.86 (59.82-82.14)	T0: 66.67 (58.33-77.08) T1: 70.83 (62.50-79.17) T2: 75.00 (66.67-80.21) T3: 70.83 (62.50-81.25) T4: 70.83 (58.33-80.21)	T0: 75.00 (66.67-83.33) T1: 75.00 (66.67-83.33) T2: 75.00 (66.67-91.67) T3: 75.00 (54.17-83.33) T4: 75.00 (58.33-75.00)	T0: 62.50) T1: 64.06 (59.39-74.22) T2: 68.75 (61.72-72.66) T3: 62.50 (54.69-71.88) T4: 62.50 (54.69-71.88)
Jones et al,[13] 2023, 2013-2021, Malaysia WHOBREF Malay version[b]	Cross-sectional study, yes	254 (preterm), 2967 (term) unspecified	0 to 16 wk after childbirth	35.67 ± 0.75 wk, 2430 ± 670g	NA	73.75 ± 13.06[a]	76.88 ± 9.31[a]	75.00 ± 0.00[a]	69.81 ± 14.00[a]
Sharma & Sohi,[14] 2007, August-December 2005, India	Cross-sectional study, yes	50 (preterm), 50 (term) mothers	Children aged 2.56 ± 1.736 mo	32.58 ± 2.976 wk, 1710 ± 576g	44.03 ± 9.13	61.13 ± 14.38[a]	61.19 ± 15.13[a]	63.19 ± 23.63[a]	64.19 ± 16.75[a]
Alves et al,[15] 2023 (mothers), November 2013-June 2015 Portugal	Cross-sectional study, yes	61 (preterm), 112 (term), mothers	4 to 6 mo after childbirth	<32 wk, NA	74.6 ± 12.5	75.9 ± 12.2	77.2 ± 14.8	75.8 ± 17.9	72.9 ± 13.9
Alves et al,[15] 2023 (fathers), November 2013-June 2015, Portugal	Cross-sectional study, yes	56 (preterm), 102 (term) fathers	4 to 6 mo after childbirth	<32 wk, NA	72.8 ± 12.4	78.3 ± 13.1	80.4 ± 13.9	74.3 ± 16.4	71.3 ± 14.6
Lee et al,[16] 2009, November 2002-April 2003, Taiwan WHOQOL-BREF-Taiwan version[b]	Cross-sectional study, yes	118 (preterm), 170 (term) unspecified	Infants aged 36 to 53 mo	NA, VLBW<1500g	NA	63.13 ± 7.48[a]	52.75 ± 10.47[a]	60.44 ± 9.72[a]	57.31 ± 8.97[a]
Wang et al,[17] 2025 (infant demise), September 2021-May 2022, USA	Cohort study, no	26, mothers	4.5 ± 2.1 y from delivery to study date	23.8 ± 1.3 wk, NA	NA	73.75 ± 19.38[a]	71.88 ± 20.00[a]	70.63 ± 23.75[a]	79.38 ± 15.00[a]

© 2026 Yip SASE et al. *JAMA Network Open*.

Wang et al,[17] 2025 (surviving infants), September 2021-May 2022, USA	Cohort study, no	32, mothers	5.1 ± 2.0 y from delivery to study date	24.1 ± 0.9 wk, NA	NA	80.00 ± 17.50[a]	81.25 ± 15.63[a]	86.88 ± 18.13[a]	86.25 ± 13.75[a]
Wolke et al,[18] 2014, January 1985-March 1986, England WHOBREF German version	Cohort study, yes	192 (mothers), 27 (fathers)	27 y old	30.4 wk, 1304g	69.6 ± 18.12	80.1 ± 14.35	74.7 ± 12.84	72.8 ± 16.61	82.3 ± 11.33

**Source, study period, country**	**Study design, comparator group**	**No. of participants, gender**	**Timing of assessment**	**Preterm infant (gestational age, birth weight), mean±SD**	**Mean±SD/median (IQR)**	
					**Physical component score**	**Mental component score**
Studies utilizing Short Form-8 Surveys (1)						
Sasaki et al,[19] 2023, June 2013-August 2016, Japan SF-8 Japanese version	Cross-sectional study, no	357, mothers	2.5 y old infants	29.2 ± 3.2 wk, NA	48.601 ± 7.552	48.593 ± 6.224
**Studies utilizing Short Form-12 Surveys (5)**						
Obregon et al,[20] 2019, May 2016-February 2017, USA	Cross-sectional study, no	50, mothers	Upon maternal discharge	31.9 ± 3.12 wk, 1796 ± 561g	49.6 ± 9.7	46.9 ± 8.9
Mautner et al,[21] 2022 (extremely preterm), December 2016-December 2018, Austria SF-12 German version	Cross-sectional study, yes	20 (extremely preterm), 20 (term) mothers	3 to 10 d postpartum	<32 wk, 1006 ± 342g	41.62 ± 7.23	36.82 ± 6.32
Mautner et al,[21] 2022 (moderately preterm), December 2016-December 2018, Austria SF-12 German version	Cross-sectional study, yes	20 (moderately preterm), 20 (term) mothers	3 to 10 d postpartum	32-37 wk, 1972 ± 294g	35.71 ± 7.43	46.58 ± 7.20
Benhammou et al,[22] 2023, 2011-2023, French regions	Cross-sectional study, no	2587, mothers	1 y after childbirth	23-34 wk, NA	51.8 ± 7.8	39.0 ± 7.7
Witt et al,[23] 2012, October 2008-July 2009, USA; SF-ver 2	Cross-sectional study, yes	297 (VLBW), 290 (NBW) mothers	When child is 5 y old	NA, VLBW<1500g	52.9 ± 8.0	48.9 ± 8.5
Suonpera et al,[24] 2023, March 2017-October 2018, England; SF-ver 1	Cross-sectional study, yes	163 (preterm), 125 (term) unspecified	When child is 11 y old	26 wk, NA	51.0 (49.6-52.4)	49.3 (47.9-50.7)
**Studies utilizing Short Form-36 Surveys (5)**						
Lee & Hsu,[25] 2012, October 2008-April 2010, USA SF36 version 2	Cross-sectional study, no	55, mothers	In NICU	27.6 ± 2.5 wk, 1023 ± 341g	-.87 ± .96	-.72 ± 1.27

© 2026 Yip SASE et al. *JAMA Network Open*.

Kamran et al,[26] 2023, January-July 2020, Iran	Cross-sectional study, no	105, mothers	In NICU	30.59 ± 2.30 wk, 1657.04 ± 485.78 g	57.42 ± 20.01	57.18 ± 19.52
Salomè et al,[27] 2022 (mothers), September 2018-September 2019, Italy	Cohort study, no	20, mothers	T1: In NICU<br>T2: 1-y post-discharge	31 ± 2.99 wk, 1375 ± 458.57g	T1: 51<br>T2: 77	T1: 32<br>T2: 65
Salomè et al,[27] 2022 (fathers), Sept 2018-Sept 2019, Italy	Cohort study, no	20, fathers	T1: In NICU<br>T2: 1-y post-discharge	31 ± 2.99 wk, 1375 ± 458.57g	T1: 80<br>T2: 84	T1: 51<br>T2: 63
Begjani et al,[28] 2025 (control), March-October 2020, Iran<br>SF-36 Persian translation	RCT, no	35, mothers	T1: In NICU<br>T2: 1 mo after intervention	30-37 wk, NA	T1:<br>Physical performance: 70.80 ± 5.27<br>Pain: 45.58 ± 8.33<br>General health: 45.96 ± 7.65<br>Limitation of physical problems: 72.34 ± 4.85<br>Total QOL: 55.80 ± 10.56	T1:<br>Mental health: 35.16 ± 5.04<br>Social function: 52.60 ± 5.85<br>Vitality: 62.12 ± 5.03<br>Limitation of emotional problems: 35.23 ± 9.27
Begjani et al,[28] 2025 (intervention), March-October 2020, Iran<br>SF-36 Persian translation	RCT, no	35, mothers	T1: In NICU<br>T2: 1 mo after intervention	30-37 wk, NA	T1:<br>Physical performance: 71.14 ± 6.16<br>Pain: 47.97 ± 6.11<br>General health: 46.73 ± 7.55<br>Limitation of physical problems: 71.45 ± 6.03<br>Total QOL: 56.13 ± 10.85	T1:<br>Mental health: 34.03 ± 5.98<br>Social function: 52.83 ± 6.60<br>Vitality: 62.80 ± 7.29<br>Limitation of emotional problems: 37.23 ± 9.18
Marthinsen et al,[29] 2024 (mothers), June 2019-March 2020, Norway<br>SF-36 Norwegian version	Cohort study, yes	T1: 16 (preterm),<br>T2: 9 (preterm),<br>T3: 10 (preterm),<br>76 (term), mothers	T1: 2 mo postpartum<br>T2: 6 mo postpartum<br>T3: 12 mo postpartum	<28-36 wk, <999g	T1: 44.97 ± 32.37<br>T2: 52.40 ± 15.52<br>T3: 49.37 ± 14.52	T1: 46.63 ± 20.15<br>T2: 45.63 ± 18.53<br>T3: 47.9 ± 29.85
Marthinsen et al,[29] 2024 (fathers), June 2019-March 2020, Norway<br>SF-36 Norwegian version	Cohort study, yes	T1: 14 (preterm),<br>T2: 6 (preterm),<br>T3: 8 (preterm),<br>76 (term), fathers	T1: 2 mo postpartum<br>T2: 6 mo postpartum<br>T3: 12 mo postpartum	<28-36 wk, <999g	T1: 50.4 ± 11.70<br>T2: 53.00 ± 3.85<br>T3: 49.4 ± 10.81	T1: 45.70 ± 22.38<br>T2: 50.73 ± 10.00<br>T3: 46.57 ± 19.85

© 2026 Yip SASE et al. *JAMA Network Open*.

Source, study period, country	Study design, comparator group	No. of participants, gender	Timing of assessment	Preterm infant (gestational age, birth weight), mean±SD)	Mean±SD/median (IQR)			
					Total score	Parent HRQOL summary score	Communication & worry summary score	Family functioning summary score
Studies utilizing PedsQL-FIM (4)								
McAndrew et al,[30] 2019 (extremely preterm), 2016-2017, USA	Cross-sectional study, yes	52 (extremely preterm), 48 (term), unspecified	T1: In NICU<br>T2: 3 mo after discharge	23-28 wk, 745g	NA	T1:<br>Physical functioning: 58 (46-75)<br>Emotional functioning: 55 (43-70)<br>Social functioning: 69 (50-88)<br>Cognitive functioning: 70 (50-90)<br>T2:<br>Physical functioning: 67 (63-79)<br>Emotional functioning: 80 (70-90)<br>Social functioning: 75 (63-88)<br>Cognitive functioning: 80 (63-98)	T1:<br>Communication: 67 (50-83)<br>Worry: 60 (43-80)<br>T2:<br>Communication: 75 (58-84)<br>Worry: 70 (55-80)	T1:<br>Daily activities: 58 (42-75)<br>Family Relationships: 75 (55-95)<br>T2:<br>Daily activities: 50 (33-71)<br>Family Relationships: 80 (65-100)
McAndrew et al,[30] 2019 (moderately preterm), 2016-2017, USA	Cross-sectional study, yes	114 (moderately preterm), 48 (term), unspecified	T1: In NICU<br>T2: 3 mo after discharge	29-36 wk, 1805g	NA	T1:<br>Physical functioning: 67 (54-79)<br>Emotional functioning: 70 (55-90)<br>Social functioning: 75 (63-88)<br>Cognitive functioning: 80 (60-95)<br>T2:<br>Physical functioning: 71 (58-81)<br>Emotional functioning: 80 (70-90)<br>Social functioning: 75 (63-94)<br>Cognitive functioning: 75 (60-95)	T1:<br>Communication: 75 (67-92)<br>Worry: 75 (55-90)<br>T2:<br>Communication: 75 (67-96)<br>Worry: 80 (70-95)	T1:<br>Daily activities: 50 (42-75)<br>Family Relationships: 90 (65-100)<br>T2:<br>Daily activities: 58 (42-75)<br>Family Relationships: 80 (70-95)
Hafeez et al,[31] 2023 (mothers), October 2021-April 2022, India<br>either Hindi or English version	Cohort study, no	46, mothers	T1: In NICU<br>T2: 3 mo follow up	30 wk, 1085g	T1: 62 (48-83)<br>T2: 71 (63-79)	T1: 64.2 (51-84)<br>T2: 72 (63-78)	Merged with parent HRQOL	T1: 64 (34-88)<br>T2: 69 (50-87)
Hafeez et al,[31] 2023 (fathers), October 2021-April 2022, India<br>either Hindi or English version	Cohort study, no	39, fathers	T1: In NICU<br>T2: 3 mo follow up	30 wk, 1085g	T1: 73 (60-86)<br>T2: 74 (65-81)	T1: 71 (60-84)<br>T2: 73 (67-84)	Merged with parent HRQOL	T1: 75 (53-89)<br>T2: 72 (57-86)
McGrath-Morrow et al,[32] 2013, January 2008-July 2011, USA	Cross-sectional study, no	69, unspecified	Mean 4.7 mo in BPD outpatient clinic	26.4 ± 2.3 wk, 872 ± 345g	69	Physical functioning: 71 ± 21<br>Emotional functioning: 84 ± 18<br>Social functioning: 81 ± 21<br>Cognitive functioning: 88 ± 15	Communication: 84 ± 19<br>Worry: 75 ± 21	Daily activities: 67 ± 29<br>Family Relationships: 84 ± 21
Peralta et al,[33] 2023, January 2006-December 2019, Switzerland	Cross-sectional study, no	533, unspecified	T1: Pre-school age children<br>T2: School age children	29 wk, 1140g	T1: 97.6 (82.6-100.0)<br>T2: 98.9 (91.7-100.0)	T1: 97.5 (85.0-100.0)<br>T2: 100.0 (91.2-100.0)	Merged with parent HRQOL	T1: 100.0 (81.2-100.0)<br>T2: 100.0 (87.5-100.0)

© 2026 Yip SASE et al. *JAMA Network Open*.

Source, study period, country	Study design, comparator group	No. of participants, gender	QOL instrument	Timing of assessment	Preterm infant (gestational age, birth weight), mean±SD)	Results
Studies utilizing miscellaneous instruments (6)						
Nordheim et al,[34] 2016, August-December 2010, Norway	RCT, no	T1: 30 (preterm), 31 (term)<br>T2: 31 (preterm), 28 (term), unspecified	Quality of Life Scale-Norwegian version	T1: In NICU<br>T2: At 3.5 y old	T1: 28.4 ± 2.7 wk, 1045 ± 248g<br>T2: 28.8 ± 3.0 wk, 1044 ± 244g	T1:<br>PreNu: 85.3 ± 12.6<br>Reference: 76.9 ± 2.6<br>T2:<br>PreNu: 88.8 ± 11.6<br>Reference: 83.1 ± 12.8
Hill & Aldag,[35] 2007 (preterm), NA, USA	Cohort study, yes	37 (pre-term), 88 (term), unspecified	MAPP-QOL	T1: Week 1 postpartum<br>T2: Week 3 postpartum	Pre-term: 24-33 wk	T1:<br>Overall: 18.9 ± 4.8<br>Psychological: 16.4 ± 7.5<br>Socioeconomic: 20.2 ± 6.1<br>Relational/spouse-partner: 23.5 ± 6.2<br>Relational/family friends: 19.6 ± 5.6<br>Health & functioning: 16.7 ± 5.4<br>T2:<br>Overall: 19.9 ± 5.3<br>Psychological: 17.9 ± 7.8<br>Socioeconomic: 20.7 ± 6.1<br>Relational/spouse-partner: 22.7 ± 6.6<br>Relational/family friends: 19.7 ± 5.7<br>Health & functioning: 19.4 ± 5.1
Hill & Aldag,[35] 2007 (near-term), NA, USA	Cohort study, yes	59 (near-term), 88 (term), unspecified	MAPP-QOL	T1: Week 1 postpartum<br>T2: Week 3 postpartum	Near-term: 33-37 wk	T1:<br>Overall: 21.0 ± 3.9<br>Psychological: 21.8 ± 5.3<br>Socioeconomic: 21.8 ± 4.7<br>Relational/spouse-partner: 22.9 ± 5.9<br>Relational/family friends: 20.6 ± 5.1<br>Health & functioning: 18.6 ± 4.3<br>T2:<br>Overall: 21.4 ± 4.8<br>Psychological: 22.5 ± 5.4<br>Socioeconomic: 21.6 ± 5.4<br>Relational/spouse-partner: 22.1 ± 6.8<br>Relational/family friends: 21.0 ± 5.2<br>Health & functioning: 20.1 ± 5.2
Petersen & Quinlivan[36] 2021, November 2009-June 2014, Brazil	Cohort study, yes	72 (preterm), 928 (term), fathers	SWLS	T1: 3rd trimester of pregnancy	NA	T2:<br>Antenatal: 27.15 ± 5.17<br>Postnatal: 27.31 ± 4.82

© 2026 Yip SASE et al. *JAMA Network Open*.

				T2: 6 wk after discharge		
Lakshmanan et al,[37] 2017 (mothers), October 2011-June 2012, USA	Cross-sectional study, no	77, mothers	ITQOL	From 6-18 mo after discharge	29.7 wk, 1169g	Total: 23.33 ± 9.63<br>Emotion: 83.00 ± 20.74<br>Time limitations: 88.00 ± 19.26
Lakshmanan et al,[37] 2017 (fathers), October 2011-June 2012, USA	Cross-sectional study, no	23, fathers	ITQOL	From 6-18 mo after discharge	29.7 wk, 1169g	Total: 27.83 ± 11.85<br>Emotion: 69.50 ± 24.44<br>Time limitations: 76.00 ± 28.15
Donohue et al,[38] 2008, July 1999-October 2000, USA	Cross-sectional study, yes	83 (preterm), 84 (term), unspecified	Quality of life inventory	12-18 mo old infants	27.89 wk, 998g	QOL: 3.58 ± 0.36
Indredavik et al,[39] 2005, November 2000-October 2002, Norway	Cross-sectional study, yes	55 (VLBW), 60 (SGA), 83 (term), unspecified	SCL-90-R	When children are 14 y old	VLBW: 28.8 ± 2.7 wk, 1174 ± 233g<br>SGA: 39.5 ± 1.1wk, 2921 ± 211g	Mothers of VLBW and SGA infants did not report more psychological symptoms than control mothers. Fathers in the VLBW group had a tendency of more phobic anxiety than fathers in the control group (P < .05), but not after exclusion of adolescents with low IQ. There were no group differences in the composite indices (data not shown).

Abbreviations: BPD, Bronchopulmonary dysplasia; CLD, Chronic lung disease; NA, Data not available or not reported in the original study; PIVH, Perinatal intraventricular hemorrhage; IUGR, Intrauterine growth restriction; ROP, Retinopathy of prematurity; SGA, small for gestational age; VLBW, Very Low Birth weight; SCL-90-R, Symptom Checklist-90-Revised; MAPP-QOL, Maternal Postpartum Quality of Life; ITQOL, Infant Toddler Quality of Life Questionnaire; MQLI, Multicultural Quality of Life Index; SWLS, Satisfaction with Life Scale; HADS, Hospital Anxiety and Depression Scale.

[a]Converted from raw score to 0-100 scale.

[b]Converted from median and IQR to mean and SD and then to 0-100 scale.

© 2026 Yip SASE et al. *JAMA Network Open*.

eTable 5. Newcastle- Ottawa Scale for Cross Sectional Studies[40,41]

Author	Year	Selection Bias Assessment (Maximum 5 stars)				Comparability (Maximum 2 stars)	Outcome (Maximum 3 stars)		Total score (Maximum 10 stars)	Quality of study[1]
		Representativeness of the sample	Sample size	Non-respondents	Ascertainment of the exposure (risk factor)	Confounding factors are controlled	Assessment of the outcome	Statistical Test		
Sharma et al.[14]	2007	1	1	0	1	1	1	1	6	Satisfactory
Lee et al.[16]	2009	1	1	0	1	2	1	1	7	Good
Moura et al.[12]	2017	1	1	1	1	2	1	1	8	Good
Neyestani et al.[8]	2017	1	1	0	1	2	1	1	7	Good
Sekhavatpour et al.[10]	2020	1	1	0	1	1	1	1	6	Satisfactory
Khanjari et al.[9]	2022	1	1	1	1	0	1	1	6	Satisfactory
Ong et al.[7]	2022	1	1	0	1	1	1	1	4	Unsatisfactory
Alves et al.[15]	2023	1	1	1	2	2	1	1	9	Very good
Jones et al.[13]	2023	1	1	0	1	2	1	1	7	Good
Li et al.[11]	2024	1	1	0	1	2	1	1	7	Good
Sasaki et al.[19]	2023	1	1	0	1	1	1	1	5	Satisfactory
Witt et al.[23]	2012	1	1	0	1	1	1	1	6	Satisfactory
Obregon et al.[20]	2019	1	1	1	0	1	1	1	5	Satisfactory
Mautner et al.[21]	2022	1	1	0	1	1	1	1	6	Satisfactory
Benhammou et al.[22]	2023	1	1	0	1	1	1	1	7	Good
Suonpera et al.[24]	2023	1	1	0	2	2	1	1	8	Good
Lee SY et al.[25]	2012	1	1	0	1	2	1	1	5	Satisfactory

© 2026 Yip SASE et al. *JAMA Network Open*.

Marthinsen et al.[29]	2024	1	1	1	1	1	1	1	7	Good
Kamran et al.[26]	2023	1	1	1	1	1	1	1	6	Satisfactory
McGrath-Morrow et al.[32]	2013	1	1	1	1	0	1	1	8	Good
McAndrew et al.[30]	2019	1	1	0	2	2	1	1	8	Good
Hafeez et al.[31]	2023	1	1	1	1	2	1	1	5	Satisfactory
Peralta et al.[33]	2023	1	1	0	1	0	1	1	7	Good
Indredavik et al.[39]	2005	1	1	1	2	2	1	1	9	Very good
Hill et al.[35]	2007	1	1	0	1	1	1	1	6	Satisfactory
Donohue et al.[38]	2008	1	1	1	1	1	1	1	7	Good
Nordheim et al.[34]	2016	1	0	0	1	1	1	1	6	Satisfactory
Lakshmanan et al.[37]	2017	1	1	1	1	2	1	1	8	Good
Petersen et al.[36]	2021	1	1	1	1	1	1	1	7	Good
Wang et al.[17]	2025	1	1	1	1	2	1	1	8	Good
Wolke et al.[18]	2014	1	1	1	2	2	1	1	9	Very good
Begjani et al.[28]	2025	1	1	1	1	1	1	1	7	Good
Salomè et al.[27]	2022	1	1	1	1	2	1	1	8	Good

[1] Unsatisfactory (0-4 points), Satisfactory (5-6 points), Good (7-8 points), Very good (9-10 points)

© 2026 Yip SASE et al. *JAMA Network Open*.

eReferences

1. World Health Organization. WHOQOL: Measuring Quality of Life. https://www.who.int/tools/whoqol/whoqol-bref

2. Vahedi S. World Health Organization Quality-of-Life Scale (WHOQOL-BREF): Analyses of Their Item Response Theory Properties Based on the Graded Responses Model. *Iran J Psychiatry*. 2010;5(4):140-153.

3. Ware JE, Sherbourne CD. The MOS 36-item short-form health survey (SF-36). I. Conceptual framework and item selection. *Med Care*. 1992;30(6):473-483.

4. Ware J, Kosinski M, Keller SD. A 12-Item Short-Form Health Survey: construction of scales and preliminary tests of reliability and validity. *Med Care*. 1996;34(3):220-233. doi:10.1097/00005650-199603000-00003

5. Varni JW, Sherman SA, Burwinkle TM, Dickinson PE, Dixon P. The PedsQL Family Impact Module: preliminary reliability and validity. *Health Qual Life Outcomes*. 2004;2:55. doi:10.1186/1477-7525-2-55

6. Kaddoura R, Zankar R, Tamim H, et al. Stress and quality of life postpreterm birth during polycrises: prospective cohort study. *AJOG Glob Rep*. 2025;5(3):100557. doi:10.1016/j.xagr.2025.100557

7. Ong SL, Soh KL, Hussin EOD, et al. Quality of life among mothers of preterm newborns in a Malaysian neonatal intensive care unit. *Belitung Nurs J*. 2022;8(2):93-100. doi:10.33546/bnj.1872

8. Neyestani A, Saeidi R, Salari M, Karbandi S. The Effect of Implementing a Discharge Program on Quality of Life of Mothers with Premature Infants. *Evid Based Care*. 2017;7(1). doi:10.22038/ebcj.2017.21095.1482

9. Khanjari S, Bell EF, Mohagheghi P, Sadeghi LS, Haghani S, Rokhsatichenijani E. The effect of family-centered education on the quality of life of the parents of premature infants. *J Neonatal Nurs*. 2022;28(6):407-412. doi:10.1016/j.jnn.2021.10.005

10. Sekhavatpour Z, Reyhani T, Heidarzade M, et al. The Effect of Spiritual Self-care Training on the Quality of Life of Mothers of Preterm Infants: A Randomized Controlled Trial. *J Relig Health*. 2020;59(2):714-724. doi:10.1007/s10943-018-0620-4

11. Li X, Lin Y, Huang L, et al. Effects of web neonatal intensive care unit diaries on the mental health, quality of life, sleep quality, care ability, and hormone levels of parents of preterm infants in the neonatal intensive care unit: A randomized controlled trial. *Intensive Crit Care Nurs*. 2024;83:103697. doi:10.1016/j.iccn.2024.103697

12. Moura MRS, Araújo CGA, Prado MM, et al. Factors associated with the quality of life of mothers of preterm infants with very low birth weight: a 3-year follow-up study. *Qual Life Res*. 2017;26(5):1349-1360. doi:10.1007/s11136-016-1456-6

13. Jones L, Mariapun J, Tan AXQ, Kassim Z, Su TT. Maternal wellbeing of Malaysian mothers after the birth of a preterm infant. *BMC Pregnancy Childbirth*. 2023;23(1):510. doi:10.1186/s12884-023-05823-y

14. Sharma M, Sohi I. Quality of Life of Mothers of Preterm and Term Babies. *J Neonatol*. 2007;21(4):281-284. doi:10.1177/0973217907021004l8

15. Alves E, Amorim M, Nogueira C, Silva S. Quality of Life of Mothers and Fathers 4 to 6 Months After Birth: The Effect of a Very Preterm Delivery. *Matern Child Health J*. 2023;27(10):1719-1725. doi:10.1007/s10995-023-03739-9

© 2026 Yip SASE et al. *JAMA Network Open*.

16. Lee CF, Hwang FM, Chen CJ, Chien LY. The interrelationships among parenting stress and quality of life of the caregiver and preschool child with very low birth weight. *Fam Community Health*. 2009;32(3):228-237. doi:10.1097/FCH.0b013e3181ab3b6a

17. Wang J, Blanchard C, Seasely A, et al. Maternal Quality of Life Following a Periviable Delivery. *Ochsner J*. 2025;25(3):162-169. doi:10.31486/toj.25.0008

18. Wolke D, Baumann N, Busch B, Bartmann P. Very Preterm Birth and Parents' Quality of Life 27 Years Later. *Pediatrics*. 2017;140(3):e20171263. doi:10.1542/peds.2017-1263

19. Sasaki H, Pak K, Mezawa H, et al. Health-related quality of life of mothers and developmental characteristics of very low birth weight children at 2.5 years of age: results from the Japan Environment and Children's Study (JECS). *Health Qual Life Outcomes*. 2023;21(1):68. doi:10.1186/s12955-023-02156-4

20. Obregon E, Litt JS, Patel P, Ziyeh T, McCormick MC. Health related quality of life (HRQoL) in mothers of premature infants at NICU discharge. *J Perinatol Off J Calif Perinat Assoc*. 2019;39(10):1356-1361. doi:10.1038/s41372-019-0463-1

21. Mautner E, Stern C, Avian A, Deutsch M, Schöll W, Greimel E. Neonates in the Intensive Care Unit: Maternal Health-Related Quality of Life and Depression After Term and Preterm Births. *Front Pediatr*. 2021;9:684576. doi:10.3389/fped.2021.684576

22. Benhammou V, Marchand-Martin L, Pierrat V, et al. Maternal health-related quality of life at 1 year after a preterm birth: role of socioeconomic status at birth. *J Epidemiol Community Health*. 2023;78(1):25-32. doi:10.1136/jech-2023-220591

23. Witt WP, Litzelman K, Spear HA, et al. Health-related quality of life of mothers of very low birth weight children at the age of five: results from the Newborn Lung Project Statewide Cohort Study. *Qual Life Res Int J Qual Life Asp Treat Care Rehabil*. 2012;21(9):1565-1576. doi:10.1007/s11136-011-0069-3

24. Suonpera E, Lanceley A, Ni Y, Marlow N. Parenting stress and health-related quality of life among parents of extremely preterm born early adolescents in England: a cross-sectional study. *Arch Dis Child - Fetal Neonatal Ed*. 2024;109(3):253-260. doi:10.1136/archdischild-2023-325429

25. Lee SY, Hsu HC. Stress and health-related well-being among mothers with a low birth weight infant: the role of sleep. *Soc Sci Med 1982*. 2012;74(7):958-965. doi:10.1016/j.socscimed.2011.12.030

26. Kamran F, Tajalli S, Ebadi A, Sagheb S, Fallahi M, Kenner C. Quality of life and stress in mothers of preterm infant with feeding problems: A cross sectional study. *J Neonatal Nurs*. 2023;29(1):68-74. doi:10.1016/j.jnn.2022.02.006

27. Salomè S, Mansi G, Lambiase CV, et al. Impact of psychological distress and psychophysical wellbeing on posttraumatic symptoms in parents of preterm infants after NICU discharge. *Ital J Pediatr*. 2022;48(1):13. doi:10.1186/s13052-022-01202-z

28. Begjani J, Fomani FK, Beiranvand F, Rajabi MM. The effect of peer-led education on the quality of life of mothers of premature infants in neonatal intensive care units: a quasi-experimental study. *BMC Res Notes*. 2025;18(1):327. doi:10.1186/s13104-025-07403-z

29. Marthinsen GN, Helseth S, Småstuen M, Bjorvatn B, Fegran L. A comparison of sleep, insomnia and health-related quality of life between mothers and fathers of preterm versus full-born infants: a longitudinal study from Norway. *Sleep Sci Pract*. 2024;8(1):8. doi:10.1186/s41606-024-00103-w

© 2026 Yip SASE et al. *JAMA Network Open*.

30. McAndrew S, Acharya K, Westerdahl J, et al. A Prospective Study of Parent Health-Related Quality of Life before and after Discharge from the Neonatal Intensive Care Unit. *J Pediatr*. 2019;213:38-45.e3. doi:10.1016/j.jpeds.2019.05.067

31. Hafeez S, Kiran S, Gupta P, Oleti TP, Saran A, Vardhelli V. Quality of Life of Parents of Very Low Birth Weight Babies Admitted to the Neonatal Intensive Care Unit. *Indian Pediatr*. 2023;60(4):317-319. doi:10.1007/s13312-023-2866-1

32. McGrath-Morrow SA, Ryan T, Riekert K, Lefton-Greif MA, Eakin M, Collaco JM. The impact of bronchopulmonary dysplasia on caregiver health related quality of life during the first 2 years of life. *Pediatr Pulmonol*. 2013;48(6):579-586. doi:10.1002/ppul.22687

33. Peralta GP, Piatti R, Haile SR, et al. Respiratory morbidity in preschool and school-age children born very preterm and its association with parents' health-related quality of life and family functioning. *Eur J Pediatr*. 2023;182(3):1201-1210. doi:10.1007/s00431-022-04783-3

34. Nordheim T, Rustøen T, Iversen PO, Nakstad B. Quality of life in parents of preterm infants in a randomized nutritional intervention trial. *Food Nutr Res*. 2016;60:32162. doi:10.3402/fnr.v60.32162

35. Hill PD, Aldag JC. Maternal perceived quality of life following childbirth. *J Obstet Gynecol Neonatal Nurs JOGNN*. 2007;36(4):328-334. doi:10.1111/j.1552-6909.2007.00164.x

36. Petersen IB, Quinlivan JA. Fatherhood too soon. Anxiety, depression and quality of life in fathers of preterm and term babies: a longitudinal study. *J Psychosom Obstet Gynaecol*. 2021;42(2):162-167. doi:10.1080/0167482X.2020.1808620

37. Lakshmanan A, Agni M, Lieu T, et al. The impact of preterm birth <37 weeks on parents and families: a cross-sectional study in the 2 years after discharge from the neonatal intensive care unit. *Health Qual Life Outcomes*. 2017;15(1):38. doi:10.1186/s12955-017-0602-3

38. Donohue PK, Maurin E, Kimzey L, Allen MC, Strobino D. Quality of life of caregivers of very low-birthweight infants. *Birth Berkeley Calif*. 2008;35(3):212-219. doi:10.1111/j.1523-536X.2008.00242.x

39. Indredavik MS, Vik T, Heyerdahl S, Romundstad P, Brubakk AM. Low-birthweight adolescents: quality of life and parent-child relations. *Acta Paediatr Oslo Nor 1992*. 2005;94(9):1295-1302. doi:10.1111/j.1651-2227.2005.tb02091.x

40. Modesti PA, Reboldi G, Cappuccio FP, et al. Panethnic Differences in Blood Pressure in Europe: A Systematic Review and Meta-Analysis. Fuchs FD, ed. *PLOS ONE*. 2016;11(1):e0147601. doi:10.1371/journal.pone.0147601

41. Wells G, Shea B, O'Connell D, et al. The Newcastle-Ottawa Scale (NOS) for assessing the quality of nonrandomised studies in meta-analyses. https://www.ohri.ca/programs/clinical_epidemiology/oxford.asp

© 2026 Yip SASE et al. *JAMA Network Open*.
