# Supplementary material for: Quality of Life of Parents of Premature Infants: A Systematic Review and Meta-Analysis
Source: JAMA Netw Open. 2026 Jan 14;9(1):e2553712. doi: 10.1001/jamanetworkopen.2025.53712 (PMC12805452; doi:10.1001/jamanetworkopen.2025.53712)
Supplement: Supplement 2. — Data Sharing Statement [file jamanetwopen-e2553712-s002.pdf]

Data Sharing Statement

Yip. Quality of Life of Parents of Premature Infants. *JAMA Netw Open*. Published January 14, 2026. doi:10.1001/jamanetworkopen.2025.53712

Data
Data available: No

Additional Information
Explanation for why data not available: This is a systematic review and meta-analysis using published data. No individual patient data were generated.
